# Synthesis and characterization of new diiodocoumarin derivatives with promising antimicrobial activities

**DOI:** 10.3762/bjoc.7.199

**Published:** 2011-12-19

**Authors:** Hany M Mohamed, Ashraf H F Abd EL-Wahab, Ahmed M EL-Agrody, Ahmed H Bedair, Fathy A Eid, Mostafa M Khafagy, Kamal A Abd-EL-Rehem

**Affiliations:** 1 Chemistry Department, Faculty of Science, Al-Azhar University, Nasr City, Cairo 11884, Egypt; 2 Chemistry Department, Faculty of Medicine, Jazan University, 82621, Jazan, Saudi Arabia; 3 Chemistry Department, Faculty of Science, Jazan University, 2097, Jazan, Saudi Arabia; 4 Chemistry Department, Faculty of Science, King Khalid University, 9004, Abha, Saudi Arabia

**Keywords:** antimicrobial, 3,5-diiodosalicylaldehyde, diethyl malonate, ethyl cyanoacetate, coumarins, Michael reaction

## Abstract

A series of 6,8-diiodocoumarin-3-*N*-carboxamides (**4**–**11**) were prepared. Treatment of ethyl 6,8-diiodocoumarin-3-carboxylate (**1**) with ethyl cyanoacetate/NH_4_OAc gave ethyl 2-(3-carbamoyl-6,8-diiodocoumarin-4-yl)-2-cyanoacetate (**12**) and 2-amino-4-hydroxy-7,9-diiodocoumarino[3,4-*c*]pyridine-1-carbonitrile (**13**), and treatment with acetone in the presence of NH_4_OAc or methylamine gave the ethyl 4-oxo-2,6-methano-2-methyl-3,4,5,6-tetrahydro-8,10-diiodobenzo[2,1-*g*]-2*H*-1,3-oxazocine-5-carboxylate derivatives **14a**,**b**. All compounds were evaluated for their antimicrobial activity and the compounds **12**–**14a**,**b** exhibited a pronounced effect on all tested microorganisms.

## Introduction

Coumarins and their derivatives are biologically and pharmaceutically interesting compounds known for their use as additives in food, perfumes, cosmetics, pharmaceuticals, platelet aggregation and agrochemicals [1–2]. Coumarins have also been reported to exhibit several biological activities, such as antimicrobial, anticancer, antifungal, anti-HIV and antioxidant properties [3–6], and they also served as versatile precursors for many organic transformations in the synthesis of a number of drug-like molecules [7–8]. Moreover, coumarin-based dyes and pigments are organic fluorescent materials exhibiting unique photochemical and photophysical properties, which render them useful in a variety of applications such as dye lasers, anion sensors, organic light-emitting diodes and solar cells [9–10].

Iodo-organic derivatives have been widely used as diagnostic-imaging drugs (such as diatrizoate meglumine, diatrizoic acid, iodipamide, iodixanol, iohexol, iomeprol and iopamidol) and as amebicides [11–12]. The benzoxazocine derivatives have received considerable attention due to their pharmacological properties, such as their antidepressant, antithrombotic, antipsychotic (for the central nervous system, CNS) and anti-breast-cancer activities [13].

In view of the important biological properties of the diiodocoumarin derivatives and iodo-organic compounds as medical agents, we planned to synthesize some new diiodocoumarin derivatives bearing side chains with different structures, as such derivatives could possess interesting and useful biological properties.

## Results and Discussion

**Scheme 1:**
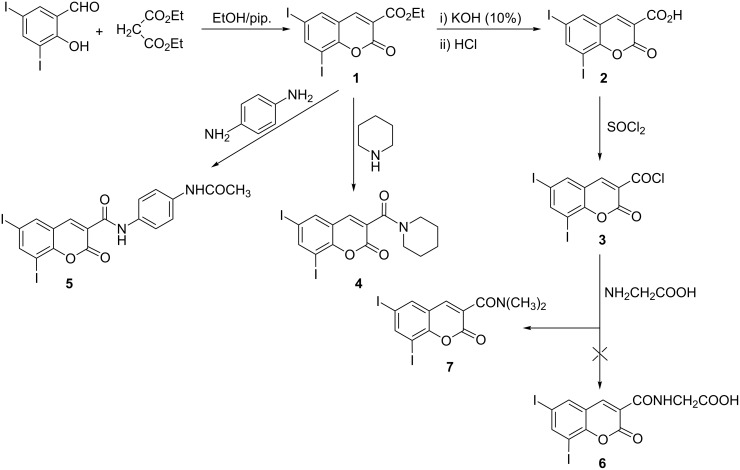
Synthesis of 6,8-diiodocoumarin derivatives **1**–**7**.

Interaction of 3,5-diiodosalicylaldehyde with diethyl malonate according to the literature procedure [14–15] afforded ethyl 6,8-diiodocoumarin-3-carboxylate (**1**). Treatment of **1** with hot ethanolic KOH (10%) followed by acidification with HCl gave the corresponding 6,8-diiodocoumarin-3-carboxylic acid (**2**), which on treatment with SOCl_2_ gave the 6,8-diiodocoumarin-3-carbonyl chloride (**3**). Treatment of **1** with piperidine in boiling ethanol or with *p*-phenylenediamine in boiling AcOH afforded the 6,8-diiodocoumarin-3-carboxamide derivatives **4** and **5**, respectively. Interaction of **3** with glycine in dry benzene under reflux gave the new 6,8-diiodocoumarin-3-*N,N*-dimethylcarboxamide (**7**) instead of 6,8-diiodocoumarin-3-ylcarbonylglycine (**6**). The formation of compound **7** suggests that two glycine molecules react with **1** followed by the loss of ammonia and decarboxylation, furnishing the observed product (Scheme 1).

**Scheme 2:**
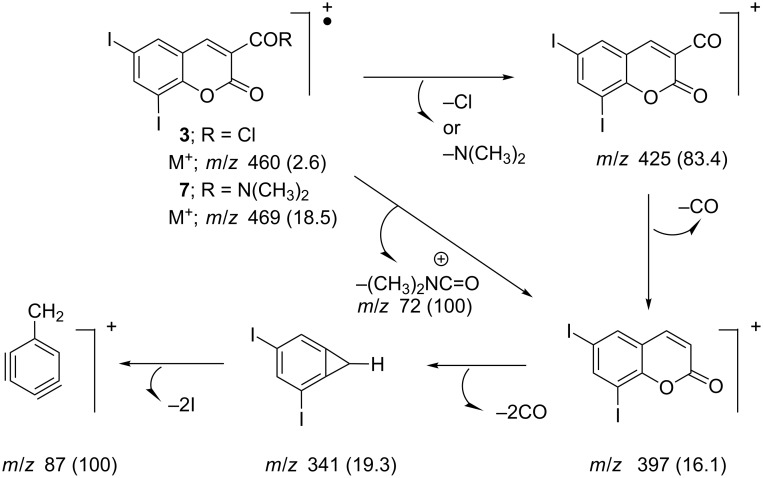
Proposed fragmentation pathways for the EI ions of the substituted 6,8-diiodocoumarins **3** and **7**.

The structures of compounds **3**–**5** and **7** were confirmed by IR, ^1^H NMR, ^13^C NMR and MS. The IR spectra for compound **3** showed 1774, 1718 cm^−1^ (2 CO); for compound **4** 1713, 1631 cm^−1^ (2 CO); for compound **5** 1718 cm^−1^ (CO); and for compound **7** 1722, 1635 cm^−1^ (2 CO). ^1^H NMR for compounds **3**–**5** and **7** showed δ at 7.64–8.70 ppm (s, 1H, H-4), and ^13^C NMR for compounds **3** and **5** showed δ at 143.2 and 147.2 ppm (C-4), respectively. The mass spectra of compounds **3** and **7** showed the corresponding molecular ion peaks at *m*/*z* 460 (M^+^, 2.6%) and *m*/*z* 469 (M^+^, 18.5%). The fragmentation pattern of compounds **3** and **7** are illustrated in Scheme 2.

**Scheme 3:**
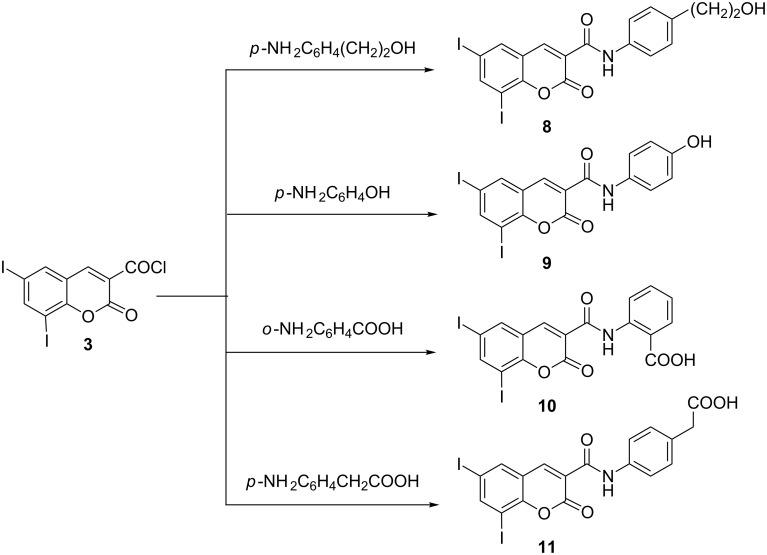
Synthesis of 6,8-diiodocoumarin-3-*N*-carboxamide derivatives **8**–**11**.

Reactions of **3** with 4-aminophenylethanol or *p*-aminophenol, or with potentially bifunctional amino acids (anthranilic acid and *p*-aminophenylacetic acid), was successful, and the corresponding 6,8-diiodocoumarin-3-carboxamide derivatives **8**–**11** were obtained (Scheme 3).

The structures of compounds **8**–**11** were established by IR, ^1^H NMR, ^13^C NMR and MS. The IR spectra of compound **8** showed 3287 cm^–1^ (OH, NH) and 1719 cm^–1^ (CO) and for compound **9** 3217 cm^–1^ (NH, OH) and 1720 cm^–1^ (CO). ^1^H NMR for **8** showed δ at 3.01 (t, *J* = 7.0 Hz, 2H, Ar-CH_2_), 3.56 (s, 1H, OH), 3.83 (t, *J* = 7.0 Hz, 2H, CH_2_–OH), and 10.49 ppm (s, 1H, NH), and for compound **11** at 3.55 (s, 2H, CH_2_), 8.71 (s, 1H, H-4), 10.10 (brs, 1H, NH), and 10.49 (s, 1H, OH). The ^13^C NMR for **11** showed δ at 160 (CO δ lactone), 163.4 (CONH), and 176.5 ppm (COOH). The mass spectra of compounds **8**–**11** provided additional evidence for the proposed structures.

**Scheme 4:**
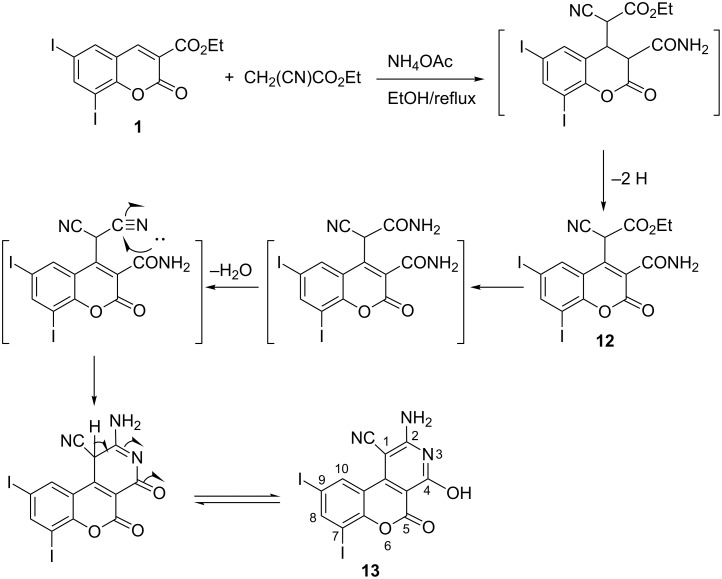
Synthesis of ethyl cyanoacetate and pyridine derivatives **12** and **13**.

As the C3–C4 olefinic bond in ethyl 6,8-diiodocoumarin-3-carboxylate (**1**) is activated by conjugation with electron-withdrawing carbonyl groups, the behavior of **1** towards activated methylene compounds under Michael reaction conditions was investigated. Thus, treatment of **1** with ethyl cyanoacetate/NH_4_OAc in boiling ethanol afforded two reaction products. The insoluble reaction product was identified as ethyl 2-(3-carbamoyl-6,8-diiodocoumarin-4-yl)-2-cyanoacetate (**12**) and the soluble reaction product was identified as 2-amino-4-hydroxy-7,9-diiodocoumarino[3,4-*c*]pyridine-1-carbonitrile (**13**), which probably formed as a result of amide formation, dehydration and intramolecular cyclization (Scheme 4).

The structures of compounds **12** and **13** were established by IR, ^1^H NMR, ^13^C NMR and MS. The IR spectra of compound **12** showed 3309, 3277 cm^–1^ (NH_2_), 2206 cm^–1^ (CN), and 1643 cm^–1^ (CO), while the ^1^H NMR for compound **13** showed δ at 7.89 (brs, 2H, NH_2_), and 9.06 (brs, 1H, OH). The spectral data of compound **13** confirmed its enol structure.

**Scheme 5:**
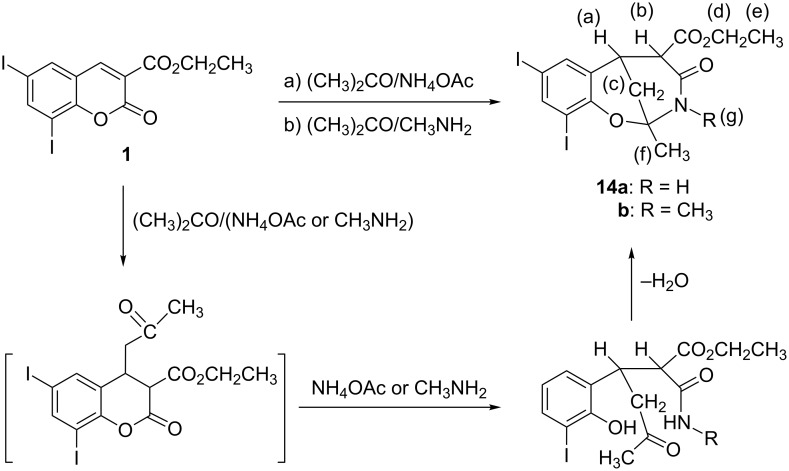
Synthesis of 1,3-oxazocine derivatives **14a**,**b**.

Reaction of **1** with acetone in the presence of NH_4_OAc or methylamine at room temperature for 7 days gave 1,3-oxazocine-5-carboxylate derivatives (**14a**,**b**) [16–18] (Scheme 5). The formation of **14** indicates that the activated methylene compounds attack at the C3–C4 olefinic bond in **1** under Michael reaction conditions to yield a cyclic Michael adduct, which underwent hydrolysis by NH_3_ or MeNH_2_ and cyclization through the elimination of H_2_O (Scheme 5).

The structure of compound **14a** was established by ^13^C NMR, which showed δ at 42.5 (CH_2_(c)), 168.4 cm^–1^ (CONH), and 170 cm^–1^ (CO). The structures of all newly synthesized compounds were confirmed by IR, ^1^H NMR, ^13^C NMR and mass spectrometry.

**Figure 1:**
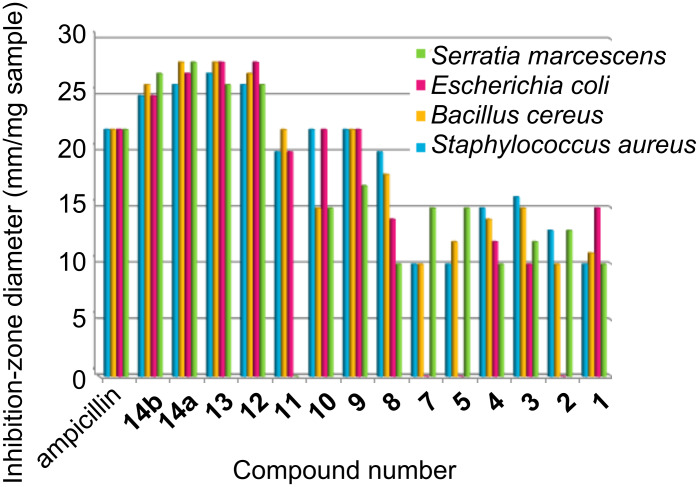
Graphical representation of the antibacterial activity of tested compounds compared to ampicillin.

**Figure 2:**
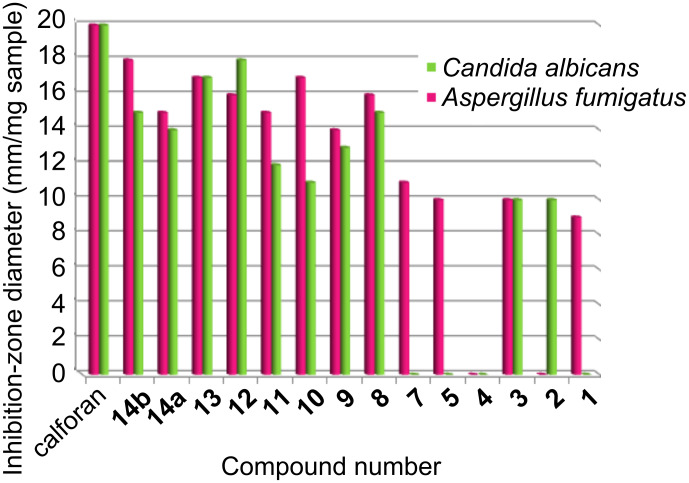
Graphical representation of antifungal activity of tested compounds, compared to calforan.

The inhibitory effects of the synthetic compounds against these organisms are given in Table 1, Figure 1 and Figure 2. Among the series tested, compounds **12**–**14a**,**b** exhibited excellent antibacterial activity, better than the standard ampicillin, against two species of Gram-positive bacteria, *Staphylococcus aureus* (NCTC-7447), *Bacillus cereus* (ATCC-14579) and two Gram-negative bacteria, *Escherichia coli* (NCTC-10410) and *Serratia marcescens* (IMRU-70), while the same compounds showed moderate antifungal activity against the tested organisms. Compounds **9**–**11** exhibited comparable activity to ampicillin against the tested bacteria and moderate to weak antifungal activity against the tested organisms. Furthermore, compounds **1**–**8** showed moderate to weak activities against all the tested bacteria and fungi, compared with the standards ampicillin and calforan. In addition, compounds **2** and **5** in the series were found to be inactive against *Escherichia coli* (NCTC-10410), while compound **11** was inactive against *Serratia marcescens* (IMRU-70). An investigation of the structure–activity relationship (SAR) revealed that the activity is considerably affected by the presence of the diiodocoumarino[3,4-*c*]pyridine, 2-methyl-8,10-diiodobenzo[2,1-*g*]-2*H*-1,3-oxazocine, diiodocoumarin-3-carboxamide or 2,3-dimethyl-8,10-diiodobenzo[2,1-*g*]-2*H*-1,3-oxazocine, and slightly decreases with the presence of different amide groups at position C-3 of the diiodocoumarin moiety or with the presence of ester, acid or acid chloride at position C-3 of the diiodocoumarin moiety.

**Table 1:** Biological activity of the newly synthesized compounds.

Compound no.^a^	Inhibition-zone diameter (mm/mg sample)					

	Gram-positive		Gram-negative		Fungi	
		
	*Staphylococcus* *aureus* (NCTC-7447)	*Bacillus* *cereus* (ATCC-14579)	*Escherichia* *coli* (NCTC-10410)	*Serratia* *marcescens* (IMRU-70)	*Aspergillus fumigatus* (MTCC-3008)	*Candida albicans* (MTCC-227)

**1**	10	11	15	10	9	–
**2**	13	10	–	13	–	10
**3**	16	15	10	12	10	10
**4**	15	14	12	10	–	–
**5**	10	12	–	15	10	–
**7**	10	10	–	15	11	–
**8**	20	18	14	10	16	15
**9**	22	22	22	17	14	13
**10**	22	15	22	15	17	11
**11**	20	22	20	–	15	12
**12**	26	27	28	26	16	18
**13**	27	28	28	26	17	17
**14a**	26	28	27	28	15	14
**14b**	25	26	25	27	18	15
Ampicillin	22	22	22	22	–	–
Calforan	–	–	–	–	20	20

^a^*c* = 1 mg mL^–1^ in DMF.

## Experimental

### General methods

Melting points were determined on a Stuart melting point apparatus and are uncorrected; IR spectra were recorded in KBr on a FT-IR 5300 spectrometer and Perkin Elmer spectrum RXIFT-IR system (ν, cm^−1^). The ^1^H NMR spectra at 300 MHz and ^13^C NMR spectra at 75 MHz were recorded in CDCl_3_ or DMSO-*d*_6_ on a Varian Mercury VX-300 NMR spectrometer. Chemical shifts (δ) are related to that of the solvent. Mass spectra were measured on a Shimadzu GMMS-QP-1000 EX mass spectrometer at 70 eV. The elemental analyses were performed at the Microanalytical Center, Cairo University, Cairo (Egypt).

**Ethyl 6,8-diiodocoumarin-3-carboxylate (1).** Ethyl 6,8-diiodocoumarin-3-carboxylate (**1**) was prepared by the interaction of 3,5-diiodosalicylaldehyde with diethyl malonate according to the literature procedures [19–21].

**6,8-Diiodocoumarin-3-carboxylic acid (2).** A solution of compound **1** (0.47 g, 10 mmol) in absolute ethanol (20 mL) was mixed with ethanolic solution of KOH (10%), which was then refluxed for 10 min. The reaction mixture was poured onto ice, acidified with HCl and recrystallized from ethanol [22].

**6,8-Diiodocoumarin-3-carbonyl chloride (3).** Compound **2** (0.44 g, 10 mmol) was dissolved in dry benzene (40 mL), 2 mL of thionyl chloride was added and the solution was refluxed for 1 h. A few drops of formic acid were added to eliminate the unreacted thionyl chloride, and the solvent was removed under reduced pressure. The solid obtained was recrystallized from benzene. Yellow crystals: Yield 92%; mp 180 °C; Anal. calcd for C_10_H_3_ClI_2_O_3_: C, 26.10; H, 0.65; found: C, 26.11; H, 0.67; IR (KBr, cm^–1^): 3055 (C–H aromatic), 1774, 1718 (2 CO); ^1^H NMR (300 MHz, CDCl_3_, δ/ppm) 8.01 (d, *J* = 1.8 Hz, 1H, Ar-H-7), 8.44 (d, *J* = 1.8 Hz, 1H, Ar-H-5), 8.58 (s, 1H, H-4); ^13^C NMR (75 MHz, CDCl_3_, δ/ppm) 86.0 (C-6), 89.1 (C-8), 118.6 (C-3), 120.3 (C-4a), 138.2 (C-5), 147.2 (C-4), 149.4 (C-7), 153.6 (C-8a), 155.0 (CO δ lactone), 162.9 (CO); MS *m*/*z* (% relative intensity): 460 (M^+^, 2.6), 459 (M – 1, 30.4), 425 (83.4), 341 (19.3), 214 (10.9), 87 (100).

**6,8-Diiodo-3-(piperidine-1-carbonyl)coumarin (4).** A solution of compound **1** (0.47 g, 10 mmol) in absolute ethanol (30 mL) was refluxed with piperidine (0.9 g, 10 mmol) for 1 h. After cooling, the solid formed was filtered off, washed with ethanol and dried under vacuum. The solid obtained was recrystallized from benzene. Colorless crystals: Yield 80%; mp 230 °C; Anal. calcd for C_15_H_13_I_2_NO_3_: C, 35.37; H, 2.55; N, 2.75; found: C, 35.36; H, 2.53; N, 2.76; IR (KBr, cm^–1^): 3040 (C–H aromatic), 2935, 2854 (C–H aliphatic), 1713, 1631 (CO); ^1^H NMR (300 MHz, CDCl_3_, δ/ppm) 1.59, 1.67, 3.29, 3.69 (m, 10H, (CH_2_)_5_), 7.64 (s, 1H, H-4), 7.78 (d, *J* = 2.1 Hz, 1H, Ar-H-7), 8.28 (d, *J* = 2.1 Hz, 1H, Ar-H-5); ^13^C NMR (75 MHz, CDCl_3_, δ/ppm) 24.30, 25.40, 48.03 (CH_2_ piperidine), 86.0 (C-8), 89.1 (C-6), 118.6 (C-3), 120.3 (C-4a), 138.2 (C-5), 147.2 (C-4), 149.4 (C-7), 153.6 (C-8a), 155.0 (CO δ lactone), 162.9 (CO-amide); MS *m*/*z* (% relative intensity): 509 (M^+^, 0.3), 424 (3.4), 341 (2.7), 214 (1.5), 84 (100).

***N*-(4-Acetamidophenyl)-6,8-diiodocoumarin-3-carboxamide (5).** A solution of compound **1** (0.47 g, 10 mmol) in glacial acetic acid (30 mL) was refluxed with *p*-phenylenediamine (1.10 g, 10 mmol) for 2 h. After cooling, the solid formed was filtered off, washed with ethanol, dried under vacuum and recrystallized from benzene. Colorless crystals: Yield 87%; mp 319 °C; Anal. calcd for C_18_H_12_I_2_N_2_O_4_: C, 37.64; H, 2.90; N, 4.88; found: C, 37.65; H, 2.92; N, 4.90; IR (KBr, cm^–1^): 3285 (NH), 1718 (CO); ^1^H NMR (300 MHz, DMSO-*d*_6_, δ/ppm) 2.40 (s, 3H, CH_3_), 7.28, 7.66 (2d, 4H, *J* = 8.4 Hz, AB-q, Ar-H), 8.37 (d , *J* = 1.8 Hz, 1H, Ar-H-7), 8.46 (d , *J* = 1.8 Hz, 1H, Ar-H-5), 8.70 (s, 1H, H-4), 8.90 (s, 1H, CH_3_CONH), 10.12 (brs, 1H, NH); ^13^C NMR (75 MHz, DMSO-*d*_6_, δ/ppm) 23.1 (CH_3_), 89.0 (C-6), 90.1 (C-8), 121.5, 128.0 (C-2', 3', 5', 6'), 114.3, 133.5, 135.6 (C-3, 1', 4'), 125.3 (C-4a), 136.2 (C-5), 143.2 (C-4), 146.4 (C-7), 148.6 (C-8a), 160 (CO δ lactone), 163.7 (CO-amide), 170.0 (COCH_3_); MS *m*/*z* (% relative intensity): 574 (M^+^, 3), 532 (M – CH_2_=C=O, 38.7), 424 (18.2), 341 (33.8), 298 (6.5), 171 (9.3), 107 (100).

**6,8-Diiodocoumarin-3-*N*,*N*-dimethylcarboxamide (7).** A solution of compound **3** (0.46 g, 1 mmol) in dry benzene (50 mL) was refluxed with glycine (0.75 g, 10 mmol) for 2 h. After cooling, the solid formed was filtered off, washed with ethanol, dried under vacuum, and recrystallized from dioxane. Colorless crystals: Yield 83%; mp 302 °C; Anal. calcd for C_12_H_9_I_2_NO_3_: C, 30.70; H, 1.92; N, 2.98; found: C, 30.72; H, 1.94; N, 3.00; IR (KBr, cm^–1^): 2931 (C–H aliphatic), 1722 and 1635 (CO); ^1^H NMR (300 MHz, DMSO-*d*_6_, δ/ppm) 2.93 (s, 3H, N-CH_3_), 2.97 (s, 3H, N-CH_3_), 8.02 (s, 1H, H-4), 8.13 (d, *J* = 2.1 Hz, 1H, Ar-H-7), 8.38 (d, *J* = 2.1 Hz, 1H, Ar-H-5); MS *m*/*z* (% relative intensity): 469 (M^+^, 18.5), 425 (83.4), 397 (16.1), 341 (19.3) and 72 (100).

### General procedure for the synthesis of 6,8-diiodocoumarin-3-carboxamide derivatives **8–11**

To a well-stirred solution of **3** (0.46 g, 1 mmol) in dry dichloromethane (DCM) containing a few drops of triethylamine (TEA) an equivalent amount of an ambient nucleophile [4-aminophenylethanol, *p*-aminophenol, anthranilic acid and *p*-aminophenylacetic acid (1.2 mmol)] was added. The reaction mixture was stirred at room temperature under dry conditions for 3 h. DCM was removed under reduced pressure until dryness, the obtained solid was then washed with 10% HCl and the remaining solid recrystallized from dioxane.

***N*-(4-(2-Hydroxyethyl)phenyl)-6,8-diiodocoumarin-3-carboxamide (8).** Yellow crystals: Yield 87%; mp 291 °C; Anal. calcd for C_18_H_13_I_2_NO_4_: C, 38.51; H, 2.32; N, 2.50; found: C, 38.52; H, 2.34; N, 2.51; IR (KBr, cm^–1^): 3287 (OH, NH), 3049 (Ar-H), 2958, 2928 (aliphatic-H), 1719 (CO); ^1^H NMR (300 MHz, DMSO-*d*_6_, δ/ppm) 3.01 (t, *J* = 7.0 Hz, 2H, Ar-CH_2_), 3.56 (s, 1H, OH), 3.83 (t, *J* = 7.0 Hz, 2H, CH_2_-OH), 7.29, 7.63 (2d, *J* = 8.2 Hz, 4H, AB-q, Ar-H), 8.37 (d, *J* = 2.0 Hz, 1H, H-7), 8.46 (d, *J* = 2.0 Hz, 1H, H-5), 8.71 (s, 1H, H-4), 10.49 (s, 1H, NH); ^13^C NMR (75 MHz, DMSO-*d*_6_, δ/ppm) 38.4 (Ar-CH_2_), 62.2 (CH_2_-OH), 111.2, 114.0 (C-6,8), 114.5, 119.8 (C-3',2',5',6'), 129.3, 132.1 (C-5,7), 135.4, 135.9, 136.0 (C-3,1',4'), 148.3 (C-4), 156.4, 159.9 (C4a,8a), 161.4 (CO δ lactone), 163.9 (CO-amide); MS *m*/*z* (% relative intensity): 561 (M^+^, 0), 543 (M – H_2_O, 3), 530 (67), 425 (M – NH-C_6_H_4_-CH_2_CH_2_OH, 100), 341 (6), 107 (36), 128 (23), 127 (14), 87 (36).

***N*-(4-Hydroxyphenyl)-6,8-diiodocoumarin-3-carboxamide (9).** Yellow crystal: Yield 85%; mp 303 °C; Anal. calcd for C_16_H_9_I_2_NO_4_: C, 36.03; H, 1.69; N, 2.63; found: C, 36.05; H, 1.68; N, 2.63; IR (KBr, cm^–1^): 3217 (NH, OH), 1720 (CO); ^1^H NMR (300 MHz, DMSO-*d*_6_, δ/ppm) 7.25–7.85 (m, 4H, Ar-H), 8.35 (d, *J* = 1.8 Hz, 1H, Ar-H-7), 8.43 (d, *J* = 1.8 Hz, 1H, Ar-H-5), 8.70 (s, 1H, H-4), 10.12 (brs, 1H, OH), 11.9 (brs, 1H, NH); ^13^C NMR (75 MHz, DMSO-*d*_6_, δ/ppm) 89.5 (C-6), 92.0 (C-8), 130.5, 124.4, 134.3, 121.5 (C-3',4',5',6'), 114.3, 140.8, 116.0 (C-3,1',2'), 125.5 (C-4a), 134.2 (C-5), 139.5 (C-4), 144.5 (C-7), 146.6 (C-8a), 160 (CO δ lactone), 163.2 (CONH), 170 (COOH); MS *m/z* (% relative intensity): 533 (M^+^, 45), 425 (M^+^ – NH-C_6_H_4_-OH, 100), 341 (16), 214 (10), 171 (17), 87 (63).

**2-(6,8-Diiodocoumarin-3-carboxamido)benzoic acid (10).** Yellow crystals: Yield 91%; mp 315 °C; Anal. calcd for C_17_H_9_I_2_NO_5_: C, 36.37; H, 1.60; N, 2.50; found: C, 36.39; H, 36.39; N, 2.52; IR (KBr, cm^–1^): 3271 (OH, NH), 3055 (Ar-H), 1751, 1651 (CO, CONH); ^1^H NMR (300 MHz, DMSO-*d*_6_, δ/ppm) 7.27, 7.64 (2d, *J* = 8.4 Hz, 4H, AB-q, Ar-H), 8.38 (d, *J* = 1.8 Hz, 1H, Ar-H-7), 8.47 (d, *J* = 1.8 Hz, 1H, Ar-H-5), 8.71 (s, 1H, H-4), 10.10 (brs, 1H, NH), 10.49 (s, 1H, OH); ^13^C NMR (75 MHz, DMSO-*d*_6_, δ/ppm) 90.0 (C-6), 92.1 (C-8), 123.0, 130.0 (C-2',3',5',6'), 114.3, 135.0, 130.5 (C-3,1',4'), 125.5 (C-4a), 133.2 (C-5), 138.5 (C-4), 144.2 (C-7), 146.1 (C-8a), 160 (CO δ lactone), 163.4 (CO-amide), 176.5 (COOH); MS *m*/*z* (% relative intensity): 561 (M^+^, 7.1), 560 (M – 1, 40.8), 517 (M^+^ – CO_2_, 2.7), 516 (M – CO_2_H, 24.5), 425 (M – NHC_6_H_4_-2-CO_2_H, 11.5), 424 (100), 341 (17), 171 (14.5) and 87 (58.2).

**2-(4-(6,8-Diiodocoumarin-3-carboxamido)phenyl)acetic acid (11).** Yellow crystals: Yield 93%; mp 285 °C; Anal. calcd for C_18_H_11_I_2_NO_5_: C, 37.57; H, 1.91; N, 2.44; found: C, 37.59; H, 1.92; N, 2.46; IR (KBr, cm^–1^): 3286 (OH, NH), 3047 (Ar-H), 2916 (aliphatic-H), 1720 (CO); ^1^H NMR (300 MHz, DMSO-*d*_6_, δ/ppm) 3.55 (s, 2H, CH_2_), 7.27, 7.64 (2d, *J* = 8.4 Hz, 4H, AB-q, Ar-H), 8.38 (d, *J* = 1.8 Hz, 1H, Ar-H-7), 8.47 (d, *J* = 1.8 Hz, 1H, Ar-H-5), 8.71 (s, 1H, H-4), 10.10 (brs, 1H, NH), 10.49 (s, 1H, OH); ^13^C NMR (75 MHz, DMSO-*d*_6_, δ/ppm) 90.0 (C-6), 92.1 (C-8), 123.0, 130.0 (C2',3',5',6'), 114.3, 135.0, 130.5 (C-3,1',4'), 125.5 (C-4a), 133.2 (C-5), 138.5 (C-4), 144.2 (C-7), 146.1 (C-8a), 160 (CO δ lactone), 163.4 (CO-amide), 176.5 (COOH); MS *m*/*z* (% relative intensity): 575 (M^+^, 12.4), 574 (M – 1, 68.9), 531 (M – CO_2_, 7.3), 425 (M – NH-C_6_H_4_-4-CH_2_COOH, 13.5), 424 (100), 341 (18.9), 171 (27.2), 106 (94.6) and 87 (59.5).

### General procedure for the synthesis of ethyl cyanoacetate and pyridine derivatives **12** and **13**

Ethanolic solution of ethyl 6,8-diiodocoumarin-3-carboxylate (**1**) (0.47 g, 10 mmol, 30 mL) was refluxed with ethyl cyanoacetate (1.13 g, 10 mmol) for 6 h. The solid precipitated was filtered off while hot, washed with ethanol and dried under vacuum, and was identified as compound **12**. The filtrate evaporated under reduced pressure to produce a solid identified as compound **13**. Compound **12** crystallized from dioxane, whereas compound **13** crystallized from chloroform.

**Ethyl 2-(3-carbamoyl-6,8-diiodocoumarin-4-yl)-2-cyanoacetate (12).** Pale yellow crystal: Yield 82%; mp 310 °C; Anal. calcd for C_15_H_10_I_2_N_2_O_5_: C, 32.62; H, 1.81; N, 5.07; found: C, 32.64; H, 1.79; N, 5.05; IR (KBr, cm^–1^): 3309, 3277 (NH_2_), 2206 (CN), 1643 (CO); ^1^H NMR (300 MHz, CDCl_3_, δ/ppm) 1.50 (t, *J* = 7.2 Hz, 3H, CH_3_), 4.44 (q, *J* = 7.2 Hz, 2H, CH_2_), 5.05 (s, 1H, CH), 8.00 (d, *J* = 1.8 Hz, 1H, Ar-H-7), 8.40 (d, *J* = 1.8 Hz, 1H, Ar-H-5), 8.70 (brs, 2H, NH_2_, exchangeable with D_2_O); MS *m*/*z* (% relative intensity): 552 (M^+^, 2), 388 (8.0), 313 (30.0), 264 (4.0), 236 (35.0).

**2-Amino-4-hydroxy-7,9-diiodocoumarino[3,4-*c*]pyridine-1-carbonitrile (13).** Pale yellow crystals: Yield 84%; mp 340 °C; Anal. calcd for C_13_H_5_I_2_N_3_O_3_: C, 30.90; H, 0.99; N, 8.32; found C, 30.92; H, 1.00; N, 8.34; IR (KBr, cm^-1^): 3374 (OH), 3277, 3228 (NH_2_), 2207 (CN), 1707, 1662 (CO); ^1^H NMR (300 MHz, CDCl_3_, δ/pm) 9.06 (brs, 1H, OH, exchangeable with D_2_O), 8.20 (s, 1H, H-8), 7.97 (s, 1H, H-10), 7.89 (brs, 2H, NH_2_, exchangeable with D_2_O); MS *m*/*z* (% relative intensity): 505 (M^+^, 100), 477 (M – CO, 18.9), 397 (20.8), 341 (18.9), 171 (25.2), 106 (32.6) and 87 (35.5).

### General procedure for the synthesis of 1,3-oxazocine-5-carboxylate derivatives **14a,b**

A mixture of compound **1** (2.35 g, 5 mmol), acetone (30 mL) and (a) ammonium acetate (0.4 g, 5 mmol) or (b) methylamine (0.16 g, 5 mmol) was stirred at room temperature for 7 days. In both cases a colorless solid formed after the solvent had evaporated under reduced pressure, and the products were identified as compounds **14a** and **14b**. The crude products were crystallized from benzene.

**Ethyl 4-oxo-2,6-methano-2-methyl-3,4,5,6-tetrahydro-8,10-diiodobenzo[2,1-*g*]-2*H*-1,3-oxazocine-5-carboxylate (14a).** Colorless: Yield 72%; mp 222 °C; Anal. calcd for C_15_H_15_I_2_NO_4_: C, 34.16; H, 2.85; N, 2.66; found: C, 34.18; H, 2.87; N, 2.68; IR (KBr, cm^–1^): 3217 (NH), 2977 (aliphatic-H), 1728, 1689 (CO); ^1^H NMR (300 MHz, DMSO-*d*_6_, δ/ppm) 0.87 (t, *J* = 6.9 Hz, 3H, CH_3_ (e)), 1.64–1.95 (m, 5H, CH_2_(c), CH_3_(f)), 3.80 (q, *J* = 4.5 Hz, 2H, CH_2_ (d)), 3.72–3.97 (m, 2H, H(a) + H(b)), 7.16 (d, *J* = 1.8 Hz, 1H, Ar-H-9), 7.92 (d, *J* = 2.1 Hz, 1H, Ar-H-7), 8.88 (brs, 1H, NH); ^13^C NMR (75 MHz, DMSO-*d*_6_, δ/ppm) 14.1 (CH_3_(e)), 24.7 (CH_3_(f)), 42.5 (CH_2_(c)), 56.0, 57.3 (CH(a)-CH(b)), 61.2 (CH_2_(d)), 130, 138, 141.9, 154 (C-2,3,5), 87.1, 87.5 (C-4,6),168.4 (CONH), 170 (CO); MS *m*/*z* (% relative intensity): 527 (M^+^, 4.3) 454 (M – CO_2_C_2_H_5_, 42.5), 182 (100), 136 (44.8), 57 (13.4).

**Ethyl 3-methyl-4-oxo-2,6-methano-2,3-dimethyl-3,4,5,6-tetrahydro-8,10-diiodobenzo[2,1-*g*]-2*H*-1,3-oxazocine-5-carboxylate (14b).** Colorless: Yield 70%; mp 198 °C; Anal. calcd for C_16_H_17_I_2_NO_4_: C, 35.50; H, 3.14; N, 2.59; found: C, 35.51; H, 3.16; N, 2.61; IR (KBr, cm^–1^): 3051 (Ar-H), 2985.6 (aliphatic-H), 1735, 1651 (CO); ^1^H NMR (300 MHz, DMSO-*d*_6_, δ/ppm) 1.23 (t, *J* = 7.2 Hz, 3H, CH_3_(e)), 1.78 (s, 3H, CH_3_(f)), 2.83 (s, 3H, NCH_3_), 2.38–2.42 (m, 2H, CH_2_(b)), 3.4–3.57 (m, 2H, H(a) + H(b)), 4.18 (q, *J* = 7.2 Hz, 2H, CH_2_(d)), 7.66 (d, 1H, Ar-H-9), 7.95 (d, *J* = 1.8 Hz, 1H, Ar-H-7); MS *m*/*z* (% relative intensity): 541 (M^+^, 3.4), 196 (59.2), 150 (27.6), 56 (100).

### Antimicrobial assays

The newly synthesized compounds were screened for their antimicrobial activities in vitro against two species of Gram-positive bacteria, namely *Staphylococcus aureus* (NCTC-7447), *Bacillus cereus* (ATCC-14579), and two Gram-negative bacteria, namely *Escherichia coli* (NCTC-10410), *Serratia marcescens* (IMRU-70); and against two species of fungi, namely *Aspergillus fumigatus* (MTCC-3008) and *Candida albicans* (MTCC-227). The tested microorganisms were obtained from the Regional Center for Mycology & Biotechnology (RCMP), Al-Azhar University.

The activities of these compounds were tested by using the disc-diffusion method [23] for bacteria and the paper-disk-diffusion method [24] for fungi. The area of zone inhibition was measured with ampicillin (30 µg mL^−1^) as the standard antibiotic reference for antibacterial activity, and calforan (30 µg mL^−1^) was used as a reference antifungal activity. The tested compounds were dissolved in *N,N*-dimethylformamide (DMF) to give a solution of 1 mg mL^−1^. The inhibition zones (diameter of the hole) were measured in millimeters (6 mm) at the end of an incubation period of 48 h at 28 °C; *N,N*-dimethylformamide showed no inhibition zone.

## Conclusion

It was interesting to note that four of the new compounds (**12**, **13** and **14a**,**b**) were found to have an antimicrobial activity greater than that of the standard antibiotic ampicillin or the standard antifungal claforan, while compounds **1**–**11** were either inactive or only weakly active against the tested microorganisms. The presence of fused diiodocoumarino[3,4-*c*]pyridine and diiodobenzo[2,1-*g*]-2*H*-1,3-oxazocine nucleus increased the antimicrobial activity, whereas the presence of diiodocoumarin-3-carboxamides decreased the antimicrobial activity.
